# Efficacy of a self-management program in patients with chronic viral hepatitis in China

**DOI:** 10.1186/s12912-019-0366-7

**Published:** 2019-09-18

**Authors:** Ying’ai Cui, Michiko Moriyama, Kazuaki Chayama, Yanhui Liu, Chunmei Ya, Basilua Andre Muzembo, Md Moshiur Rahman

**Affiliations:** 1 Department of Chronic Care and Family Nursing, Graduate School of Biomedical & Health Sciences, Hiroshima University, 1-2-3 Kasumi, Minami-ku, Hiroshima, 734-8553 Japan; 2 Department of Gastroenterology and Metabolism, Graduate School of Biomedical Sciences, Hiroshima University, 1-2-3 Kasumi, Minami-ku, Hiroshima, 734-8553 Japan; 3 School of Nursing of Tianjin University of Traditional Chinese Medicine, 88 Yuquan Road, Nankai District, Tianjin, People’s Republic of China 300193; 4 Department of Infection Prevention, Tianjin Second People’s Hospital, 75 South Causeway Road, Nankai District, Tianjin, People’s Republic of China; 5 Department of Public Health, School of Medicine, International University of Health and Welfare, Narita campus 4-3, Kozunomori, Narita-shi, Chiba-ken 286-8686 Japan; 6 Graduate School of Biomedical & Health Sciences, Hiroshima University, 1-2-3 Kasumi, Minami-ku, Hiroshima, 734-8553 Japan

**Keywords:** Efficacy, Self-management, Patient education, Hepatitis, Telenursing, Quality of life, China

## Abstract

**Background:**

Chronic hepatitis, mainly B or C, increases the risk of hepatocellular carcinoma and remains an emerging issue in the globe. China has high rates of liver cancer incidence and mortality in the world. To address such challenges, adequate management of chronic hepatitis is required. Self-management education is one alternative for improving the hepatitis patients’ knowledge of the disease, mental health, and clinical management.

This study aimed to examine the quality of life (QOL), psychological effects, and behavioral changes of a self-management program which allows continuity of care for chronic hepatitis B and C patients.

**Method:**

In a six-month, randomized controlled trial, we invited 73 chronic hepatitis B/C inpatients to receive (i) two face-to-face education sessions provided by a nurse during hospitalization, and monthly telephone counseling at home after discharge; (ii) or usual care treatment (control group). The primary endpoint (patients’ QOL) and secondary outcomes (including self-efficacy, depression symptoms, perceived cognition of illness and behavioral changes) were assessed. In addition, we conducted qualitative data analysis to facilitate the evaluation of the interventions.

**Results:**

Sixty (82.2%) out of 73 eligible patients with chronic hepatitis B/C (aged 34.9 ± 8.9 years) participated in the study. The intervention group (*n* = 30) significantly improved on outcomes including QOL, self-efficacy, perceived cognition of illness, and behavioral changes, whereas the control group significantly decreased their healthy behaviors. In terms of behavioral changes, alcohol avoidance, dietary adherence, and stress management also improved in the intervention group. However, there were no significant improvements in symptoms of depression. Most participants (80%) in the intervention group stated that they benefited from the program.

**Conclusions:**

This program contributed to patients’ acquisition of self-management skills to cope with their illnesses, and significantly improved their QOL. This program serves as a reminder for nurses who care for patients with chronic viral hepatitis to acquire these skills as it would help them address the daily needs of their patients.

**Trial registration:**

UMIN000025378. Registered December 23, 2016.

**Electronic supplementary material:**

The online version of this article (10.1186/s12912-019-0366-7) contains supplementary material, which is available to authorized users.

## Background

In the year 2015, there were about 325 million people infected with hepatitis B (HBV) or C (HCV) worldwide [1]. Research shows that chronic HBV or HCV infection increases the risk of hepatocellular carcinoma [2]. China has the highest number of deaths due to hepatocellular carcinoma in the world, particularly in 2012, where the mortality rate as a result of this disease was as high as half of the globally-reported cases [3]. Notably, liver cancer was the second main cause of death among all types of cancer in China in 2015 [4]. Therefore, reducing the incidence of liver cancer in China alone could widely decrease the number of disease-related deaths around the globe.

In patients with chronic hepatitis B or C, antiviral therapy is recommended for treatment and prevention of cirrhosis and hepatocellular carcinoma [5]. The outcomes of antiviral therapy depend on several factors including viral load, regular follow-up and adherence to medication, and lifestyle issues such as alcohol consumption [5].

In China, patients with chronic hepatitis B or C are likely to have insufficient information regarding antiviral therapy and the existence of counseling related to lifestyle changes. A significant number of these patients are reluctant to receive/continue treatment and rely on folk remedies that can make the disease worse [6]. In addition, some antiviral therapies for chronic hepatitis, such as interferon-based therapies, had been reported to have adverse side effects such as anxiety and depressive disorders [7]. Moreover, most patients with chronic hepatitis B or C requiring long-term therapy are at increased risk of having financial problems and suffering from mental stress [8]. Discrimination against viral hepatitis is strong and the social stigma that is attributed to this disease provides a negative attitude towards compliance on treatment [9]. All these factors, including the stigma of infection, may reduce patient’s quality of life (QOL) and treatment adherence that can contribute to life-threatening complications.

The rationale for using self-management education for chronic disease management is that it provides patients with tools to use in coping with their diseases such as motivation for changing lifestyles, increase problem-solving skills, and engaging the patients in the day-to-day management of their illness [10]. Therefore, self-management education is an important approach to increase adherence to antiviral therapy [11]. For example, in a systematic review of fourteen HBV and/or HCV studies related to patient education, nurse-led sessions, significantly improved patients’ knowledge of their adherence to treatment [11]. In another study, self-management education significantly improved patients’ QOL and depression at the end of the program, and these effects had been sustained 1 year after the intervention ended [12].

Specifically, studies on self-management education regarding hepatitis in China found improvement in the QOL and self-efficacy [13, 14]. However, the research approaches used in these studies were limited to knowledge provision. Research shows that non-adherence to antiviral treatment for HBV or HCV is frequent in China [15]. Thus, it is obvious that a lack of self-management education during hospitalization for HBV or HCV, and following discharge in China had failed to address adherence problems in patients under treatment for HBV or HCV. Therefore, in our program, we invested more effort into mental health issues and started to implement the program from the hospitalization stage, and continued our education efforts after discharge, thus increasing patients’ engagement in the program, and leading to reduced anxiety and dropout prevention.

This study examined the QOL, psychological effects, and behavioral changes of a self-management program which allows continuity of care from hospital to community for chronic hepatitis B and C patients.

### Theoretical background and framework of the program

**Fig. 1:**
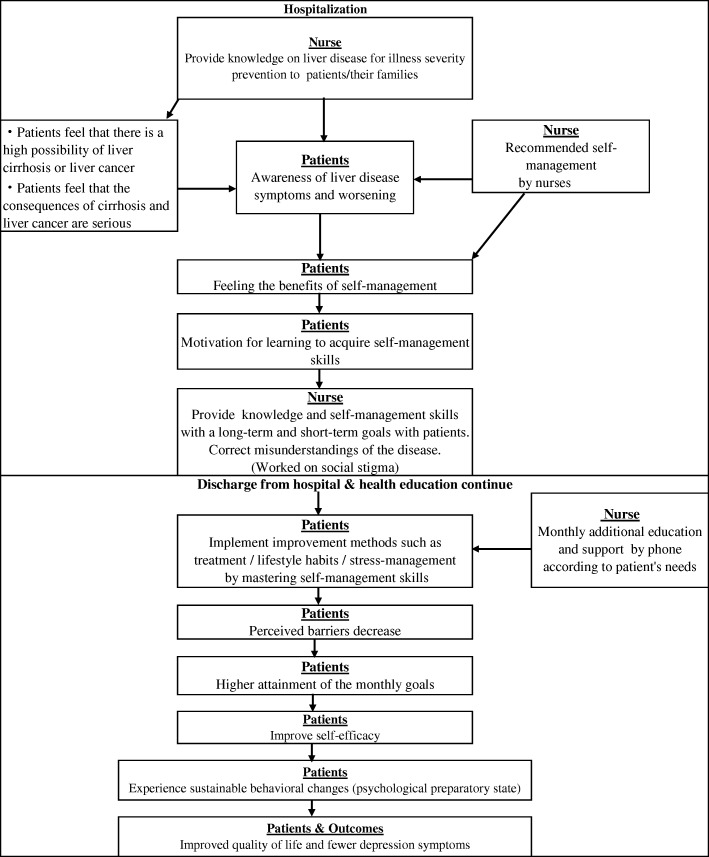
Conceptual framework of the program

This educational program (Fig. 1) aimed to help patients acquire self-management skills for long-term management of their illness and improve their QOL in the community after being discharged from hospital. We hypothesized that after providing chronic hepatitis self-management education to the patients, cognition (i.e. misunderstanding of the disease related to its social stigma) would be corrected, lifestyle behavior would change, and self-efficacy would improve as monthly goals for them to attain. This educational program aimed to help patients acquire self-management skills for long-term management of the disease. Our operational definition of self-management education in this study was in line with one from a previous research strategy by Moriyama and colleagues [16]. In this study, we used the self-management education approaches for supporting a process involving patients’ understanding of their disease through the establishment of a partnership between patient and nurse. This program also included the provision of support by healthcare professionals and the patient’s family members, providing relevant information, understanding the basics of decision-making and the treatment regimen, appropriate management of lifestyle and emotions, and sustenance of health management activities of the patients form the hospital nurses.

In addition, for effective patients’ motivation, we used the Health Belief Model [17, 18], provided sufficient information about the disease, and explained laboratory test results, forecasting probable threats, and discussed the benefits and barriers of behavioral changes in daily life. Self-efficacy was strongly related to behavioral modification; i.e. we used small step methods in which the patients and the researcher developed action plans and evaluated how effectively they had been implemented on a monthly basis.

We provided more thorough education on stress-management because of the nature of the disease and side effects of the treatment. Adequate knowledge of the disease and infection control, anxiety control, and problem-solving were taught thoroughly to deal with the social stigma, isolation [9], and strong anxiety [7]. Issues related to the social stigma were also discussed.

## Methods

### Trial design

This was a stratified sampling, open-label, and controlled trial, retrospectively registered at http://www.umin.ac.jp/ under the following project identifiers, IDs: UMIN000025378. We decided to conduct an open-label, and controlled trial because of the difficulties we experienced in blinding both researchers and participants to the interventions.

### Participants

The inclusion criteria were: a) patients diagnosed with chronic hepatitis B or C by a physician and requiring antiviral therapy; b) adult aged ≥18 years; c) patient who could be contacted by telephone after discharge. Patients previously diagnosed with depression, cognitive impairments (Mini Mental State Examination ≤27) [19], illiterate patients or those suffering from other types of hepatitis (i.e. physician-diagnosed liver diseases not due to HBV or HCV), were excluded from the study.

### Sample size

For reliable detection, study groups of 46 participants and a difference between the groups in mean scores of the Chronic Liver Disease Questionnaire (CLDQ) of 33.1 [(standard deviation 23.6, mean CLDQ scores 170 versus 30.6, mean CLDQ scores of 136.9 [20], alpha of 0.05 (two-tailed), and power of 90%)] were needed. Assuming that 20% of the participants would be lost to follow-up, the sample size was set at 30 for each group and 60 for the 2 groups combined.

### Recruitment facility

Between August to October 2016, patients were recruited from Tianjin Infectious Diseases Hospital, China, by a researcher at the inpatient hepatitis department. This is a teaching hospital and one of the largest three-level first-class infectious disease facilities, specializing in hepatitis treatment.

### Randomization

After taking written consent, the participants were stratified by the type of hepatitis to avoid influencing the results, and then randomly assigned to an intervention or usual care (UC) group. The assignee in-charge was not involved in patient education. Participants were allocated according to the last number on the patient’s chart: even numbers were placed into the intervention group, and odd numbers into the UC group. The program was implemented from August 2016 to May 2017.

### Self-management education for the intervention group

The program started after hospitalization and was completed six-months after discharge, which is the minimum duration for behavioral modification [21].

During hospitalization, participants received two face-to-face individual educational sessions that lasted 30 to 60 min from the principal researcher (a nurse) immediately after hospitalization and the day before discharge. After discharge, they received 20- to 40- min telephone education sessions once per month for 6 months by the researcher.

During the first session, the researcher identified risk factors by assessing the participant’s laboratory data, dietary habits and daily activities, psychosocial information, and physical conditions. Then the researcher taught the self-management program using a specially-developed booklet and notebook in which participants recorded weekly behaviors on medication and dietary adherence, prevention of fatigue, quitting smoking, alcohol avoidance, and stress management.

The researcher and the participants worked together in setting short-term goals (action plans related to management of diet, exercise, and other daily life activities) for the following month to achieve a long-term goal of this program (a reason why he/she committed to this program).

### Education for the UC group

The UC group received the standard education implemented by the hospital nurses. In addition, the researcher also provided the same self-management booklet and the notebook and explained how to use them. Participants received routine standard medical consultations.

### Quality assurance of intervention materials and intervention implementers

The booklet used in the study covered the contents of the patient’s education, according to the self-management and lifestyle recommendations of the evidence-based clinical practice guidelines for hepatitis B and C. The content was created based on studies of hepatitis patients and discussions with a hepatologist and chronic care specialist. To ensure quality, the researcher received training sessions on self-management and motivation as interviewing skills.

### Evaluation and data collection

#### Quality of life

To evaluate the effect of the program, a change in their score on the Chronic Liver Disease questionnaires, a modified Chinese version [22] (CLDQ) (Cronbach’s alpha = 0.83) was set as the primary endpoint. The questionnaire consists of 29 items and responses are made on a 7-point scale, scores ranging from 29 to 203, with higher scores indicating a better QOL. These questions operationalize QOL as fatigue, activity, emotional function, abdominal symptoms, systemic symptoms, and worry.

#### Symptoms of depression

Depression was assessed with 20 items using the Center for Epidemiologic Depression scale in the Chinese population [23] (CES-D) with the McDonald’s omega hierarchical coefficient estimated at 0.855. Participants having a score of ≥16 are considered to be suffering from depression.

#### Self-efficacy

Self-efficacy was defined as the confidence in the ability to perform self-management activities including the confidence to cope with chronic hepatitis viral medication and dietary adherence, side effects, prevention of fatigue, and communication with the healthcare providers in the context of chronic viral hepatitis. It was measured using the General Self Efficacy Scale (GSES), a modified Chinese version, that has 10 items [24] (Cronbach’s alpha = 0.91). The score ranged from 10 to 40 points, with higher scores indicating better confidence.

#### The cognition to illness and cognition to behavioral change

The changes in perceived cognition regarding illness and self-management based on the Health Belief Model [18] were evaluated by a 5-point rating scale. Perceived susceptibility refers to one’s subjective perception of the risk of contraction where point 1 indicated “disagree completely” and 5, “agree completely”. Perceived severity is a feeling concerning the seriousness of illness with 1 meaning “do not feel serious at all” and 5, “very serious”. Perceived benefit is one’s assessment of the value or efficacy of engaging in a health-promoting behavior to decrease the risk of disease, and point 1 meaning “no meaning” and 5, “extremely meaningful”. Perceived barrier is one’s assessment of the barriers to behavioral change where point 1 indicated “very difficult” and point 5 meant “not at all difficult” (Additional file 1).

#### Health behavior changes

We asked about the patients’ monthly behavioral changes in medication, dietary habit, prevention of fatigue, quitting smoking, alcohol avoidance, and stress management recorded in the notebook. The responses were provided on a 6-point rating scale where 1 meant “not carried out”; 2, “carried out sometimes”; 3, “carried out once a week”; 4, “carried out 2 to 3 days a week”; 5, “carried out 4 to 5 days a week”; and 6 meant “carried out every day” (Additional file 1).

#### Qualitative program evaluation

The evaluation of the program was conducted in the intervention group at the end of the program by mail. Using a structured and semi-structured questionnaire, participants in the intervention group were asked about their impressions regarding the self-management education.

In terms of our data collection procedures, baseline data that included participant’s characteristics and subject profiles were obtained from medical records and face-to-face interviews upon enrollment. Psychological indicators of QOL, depression, and self-efficacy, and the cognition of illness, and health behavior were obtained upon enrollment. The psychological indicators were assessed at 3 and 6 months, and the illness cognition questionnaire was obtained at 6 months after discharge by mail. Behavioral change data were obtained from the intervention group during phone calls conducted each month, whereas for the UC group they were collected at 6 months after discharge based on patients’ notebooks to avoid affecting the results.

### Data analysis

Baseline data were analyzed using chi-square test, t-test or Mann-Whitney U-test. For evaluation of the psychological indicators at 3 and 6 months between the two groups, two-way repeated measures ANOVA and multiple comparisons (Bonferroni’s correction) were conducted. Analysis of covariance (ANCOVA) with gender as covariate was also conducted to eliminate a potential gender bias. To compare the changes in the number of participants who were suspected to have depression at baseline and at the 6th month after discharge, difference-in-differences (D-I-D) model was carried out, and the *p* value was calculated by Fisher’s exact test for numeric data.

Mann-Whitney U-test and ANCOVA with gender as covariate were performed to assess the changes in cognition and behaviors at baseline and 6 months after discharge. Data were analyzed using SPSS ver. 22.0 (IBM Co., Armonk, NY), and the D-I-D model was performed with Easy R (EZR) on R commander ver. 1.36, and the significance level was set at the level of *p* < 5%. Qualitative data were descriptively analyzed, and symbolic expressions were extracted from the semi-structured questionnaire.

## Results

### Baseline characteristics

**Fig. 2:**
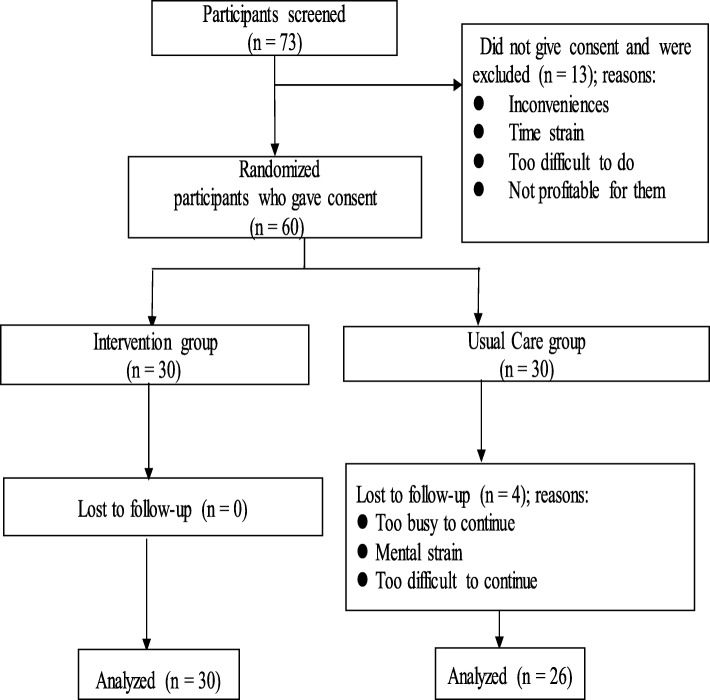
Recruitment, randomization and completion flowchart

A total of 73 participants were screened and 60 of them agreed to participate in the study: intervention group (*n* = 30) and UC group (*n* = 30). All participants in the intervention group completed the study; however, 4 participants dropped out of the UC group (Fig. 2). The mean age of all the participants was 34.9 ± 8.9 years, and 45% (27/60) of them had a history of hospitalization due to hepatitis. The treatment regimens received by the participants were not different between the two groups. A total of 86.7% (52/60) of the participants had hepatitis B, whereas hepatitis C was found in 13.3% (8/60) of the participants (Table 1).

**Table 1:** Baseline characteristics and participant profiles

Variable	Intervention group	Usual care group	*p* value
	(*n* = 30)	(*n* = 30)	
Characteristics	n (%) or	n (%) or	
	mean ± SD	mean ± SD	
Females	15 (50.0)	6 (20.0)	0.029^a*^
Employed	25 (83.3)	22 (73.3)	0.532^a^
Marital status: married	26 (86.7)	26 (86.7)	1.000^a^
Living with another person	28 (93.3)	27 (90.0)	1.000^a^
Age (years)	33.2 ± 7.29	36.6 ± 10.56	0.152^b^
Subject profiles			
Comorbidity	25 (83.3)	22 (73.3)	0.532^a^
Diabetes mellitus	27 (90.0)	28 (93.3)	1.000^a^
Recurrent history of liver disease in the past 2 years	19 (63.3)	14 (46.7)	0.299^a^
Use of insulin	29 (96.7)	28 (93.3)	1.000^a^
Treatment regimen			
Antiviral medicine	11 (36.7)	9 (30.0)	0.785^a^
Anti-fibrosis therapy	13 (43.3)	15 (50.0)	0.796^a^
Immunotherapy	4 (13.3)	6 (20.0)	0.731^a^
Liver protection therapy	27 (90.0)	28 (93.9)	1.000^a^
Peg interferon	11 (36.7)	14 (46.7)	0.601^a^
Types of hepatitis			
Hepatitis B	26 (86.7)	26 (86.7)	1.000^a^
Hepatitis C	4 (13.3)	4 (13.3)	

*SD* Standard Deviation

*p <  0.05

^a^ *p* value is based on Pearson’s chi-square test

^b^ *p* value is based on t-test

### Efficacy of this program

#### QOL

There were significant differences in QOL scores between the intervention and UC group (*p* <  0.001). The antagonistic interaction between the intervention and UC group was also significant (*p* <  0.001). Multiple comparisons (Bonferroni’s correction) were conducted between two groups, and significant differences were observed at the 3rd (*p* = 0.001) and 6th month (*p* <  0.001). The scores of QOL in chronic hepatitis patients improved from 135.2 to 164.0 in the intervention group; however, the scores decreased from 140.0 to 133.5 in the UC group. The ANCOVA results also showed a significant difference between the two groups at the 3-month (*p* = 0.001) and 6-month (*p* <  0.001) assessments (Table 2).

**Table 2:** Change in mean scores of CLDQ, CES-D and GSES in the intervention and usual care groups at 3 and 6 months follow-up

Indicator	Time point	Two-way repeated measures analysis of variance (ANOVA)									Analysis of covariance (ANCOVA)*		
		IV group			UC group			*p* value					
		n	Mean	SD	n	Mean	SD	Interaction	Within group	Between group	I - J	SE	*p* value**
CLDQ (QOL scale)													
	Baseline	30	135.2	20.6	26	140.0	21.2	< 0.001	< 0.001	< 0.001	−3.893	6.053	0.523
	3 M	30	152.0	14.5	26	136.5	17.7				16.230	4.652	0.001
	6 M	30	164.0	13.5	26	133.5	14.2				30.544	4.026	< 0.001
CES-D (Depression scale)													
	Baseline	30	16.7	8.9	26	12.4	7.8	0.002	< 0.001	0.537	3.891	2.439	0.117
	3 M	30	11.8	6.3	26	11.8	5.8				−0.267	1.749	0.879
	6 M	30	9.5	5.1	26	11.0	5.3				−2.203	1.499	0.148
GSES (Self – efficacy scale)													
	Baseline	30	2.4	0.6	26	2.5	0.6	< 0.001	0.005	0.001	−0.144	0.177	0.422
	3 M	30	2.7	0.5	26	2.3	0.4				0.434	0.133	0.002
	6 M	30	3.1	0.4	26	2.2	0.4				0.843	0.118	< 0.001

*CLDQ* Chronic Liver Disease questionnaires, *CES-D* the Center for Epidemiologic Depression scale, *GSES* General Self Efficacy Scale, *IV* Intervention group, *SD* Standard Deviation, I – J = Difference in average value, *SE* Standard Error, *ANOVA* Analysis of variance, *ANCOVA* Analysis of covariance, *UC* Usual care group, *BL* Base line, *M* month

*Analysis of covariance was carried out using gender as a covariate

**p values comparing the intervention group with the control group were obtained by multiple comparisons (Bonferroni’s correction)

#### Depression symptoms

There was no significant difference in pairwise comparisons between the two groups (*p* = 0.537); however, the interaction was significant (*p* = 0.002). The multiple comparisons (Bonferroni’s correction) between the two groups showed no significant differences at the 3rd (*p* = 0.969) and 6th month (*p* = 0.267). However, in the intervention group, the respondents’ symptoms of depression scores (CES-D) decreased from little (16.7) to none (9.5) and also decreased from 12.4 to 11.0 in the UC groups (Table 2). Although there was no statistical significance (*p* = 0.701) in differences in the number of participants who were suspected to have depression, the number decreased dramatically from 13 to 5 in the intervention group, compared with 8 to 5 in the UC group (Table 3).

**Table 3:** Depressive tendency from baseline to the 6th month after discharge

Group (n)	Baseline	6th month	OR	95% CI	*p* value*
	n (%)	n (%)			
IV (30)	13 (43.3)	5 (16.7)	1.60	0.27–9.61	0.701
UC (26)	8 (30.8)	5 (19.2)	Reference	Reference	Reference

*IV* Intervention group, *UC* Usual care group, *CI* Confidence interval

*p value is based on Fisher’s exact test for count data

#### Self-efficacy

There were significant differences found in pairwise comparisons between the two groups (*p* <  0.001), and the interaction was also significant (*p* <  0.001). Multiple comparisons (Bonferroni’s correction) between the two groups showed significant differences at the 3rd (*p* = 0.001) and 6th month (*p* <  0.001). The confidence scores (GSES) in the intervention group improved from 2.4 to 3.1 whereas it decreased from 2.5 to 2.2 in the UC group. The ANCOVA results also showed a significant difference between the two groups at 3 (*p* = 0.002) and 6 months (*p* <  0.001) (Table 2).

### The perceived cognition of illness and behavioral change

Perceptions of susceptibility and severity of illness were not significant in the intervention and UC groups (*p* = 0.170 and *p* = 0.057, respectively). Perceived benefit for behavioral change (median score) was 4.0 in both groups at the baseline, and motivation was maintained from 4.0 to 4.0 in the intervention group; in comparison, it decreased from 4.0 to 3.0 in the UC group. Perceived barriers were 3.0 in both groups at the beginning. After the program, the barrier score improved to 4.0 in the intervention group and decreased to 2.5 in the UC group. Changes in benefit and barriers were statistically significant (*p* <  0.001) in both groups. After adjusting for confounding factors, the ANCOVA result showed that there was a significant difference in perceived severity (*p* = 0.036), perceived benefit (*p* <  0.001), and perceived barriers (*p* <  0.001) between the two groups (Table 4).

**Table 4:** Changes in perceived cognition of illness at the 6th month after discharge in the intervention and usual care groups

Mann-Whitney U-test								Analysis of covariance		
Measure			Baseline		6th month			(ANCOVA)		
		n	Median	Interquartile range	Median	Interquartile range	*p* value	I - J	SE	*p value*
Perceived susceptibility	IV	30	3.5	1–5	4.5	2–5	0.170	0.517	0.297	0.087
	UC	26	3.5	2–5	4	2–5				
Perceived severity	IV	30	4	4–5	5	4–5	0.057	0.332	0.154	0.036
	UC	26	4	4–5	5	3–5				
Perceived benefit	IV	30	4	3–4	4	3–5	< 0.001	1.180	0.185	< 0.001
	UC	26	4	3–5	3	2–4				
Perceived barriers	IV	30	3	1–4	4	1–5	< 0.001	1.233	0.245	< 0.001
	UC	26	3	2–4	2.5	1–4				

*SE* Standard Error, *IV* Intervention group, *UC* Usual care group, I – J = Difference in average value

### Behavioral changes

All participants (100%) completed recording in the notebooks in the intervention group; whereas only 73% recorded their behaviors in the UC group. With respect to behavioral changes, alcohol avoidance (*p* = 0.001) improved in the intervention group after 6 months. After adjusting for confounding factors, ANCOVA results showed that there was a significant difference between the two groups in perceived dietary habit (*p* = 0.034), alcohol avoidance (*p* <  0.001), and stress management (*p* = 0.037) (Table 5).

**Table 5:** Changes in health behaviors at the 6th month after discharge in the intervention and usual care groups

Mann-Whitney U-test									Analysis of covariance		
Measure			Baseline			6th month			(ANCOVA)		
		n	Median	Interquartile range	n	Median	Interquartile range	*p* value	I - J	SE	*p* value
Alcohol avoidance	IV	30	6	1–6	30	6	4–6	0.001	0.910	0.210	< 0.001
	UC	26	5	3–6	19	4	4–6				
Dietary habit	IV	30	5	2–6	30	5	3–6	0.090	0.556	0.254	0.034
	UC	26	5	3–6	19	4	3–6				
Medication	IV	30	6	5–6	30	5	2–6	0.298	−0.275	0.284	0.338
	UC	26	6	3–6	23	5	3–6				
Prevention of fatigue	IV	30	5	2–6	30	5	2–6	0.564	0.051	0.362	0.888
	UC	26	5	3–6	19	4	2–6				
Smoking cessation	IV	30	6	1–6	30	6	1–6	0.134	−0.054	0.527	0.919
	UC	26	5	1–6	19	5	1–6				
Stress management	IV	30	6	1–6	30	5.5	3–6	0.065	0.579	0.270	0.037
	UC	26	5	3–6	19	5	4–6				

*SE* Standard Error, *IV* Intervention group, *UC* Usual care group, I – J = Difference in average value

### Qualitative evaluation of the program

All participants in the intervention group evaluated the program, where 80 % evaluated it as “very good,” and “good”, and agreed that “this type of education is necessary.” Regarding the program period, 40% stated that it was “appropriate.” The program content was judged by 83.3% as being “appropriate”, whereas more than 90% evaluated the face-to-face and telephone counseling as “very good” and “good.”

Regarding the booklet, more than 83.3% answered that “they used it and had read the whole booklet,” and 83.4% reported that “it was useful and helped their understanding.” In terms of usage frequency of the booklet, 93.4% stated that they used it daily.

Additionally, participants evaluated our education style. One of the participants commented that:

*This is the first time I have come to contact with this type of Education. [ … ] I feel interesting. Set a goal by myself, did little by little, I did enjoy accomplishing on it.*

This education mostly focused on stress management and treating social stigma. One participant provided the following comment:

*I was very nervous when the nurse first talked to me [ … ]. After a long period of nurse’s contact, my nervousness disappeared. I knew that the nurse slowly helped me adjust the lifestyle that suits my illness.*

The social stigma had a strong effect on participants as even a telephone call made the subjects worry. One participant stated:

*I still don't want to tell about my disease to people around me. So it would be better to contact me using other ways (not telephone call). For example, mailbox, etc.*

*I didn’t want to carry the booklet, because it was obvious that “hepatitis” is on the cover page. This disease is still very sensitive in our country. You better change the cover.*

## Discussion

In this study, we developed an educational program to encourage the acquisition of self-management skills and improve QOL for patients affected with chronic hepatitis B or C and evaluated the program efficacy. Our findings revealed that self-management intervention significantly improved QOL and self-efficacy of the participants. This improvement suggests that the self-management education program was a process and applying motivational interviewing techniques supported and empowered the participating patients. Being with patients and spending time with them along with some support and care to solve problems related to their diseases might be effective to alleviate disease-related distress. This is a crucial finding because improving patient’s self-efficacy can reduce distress associated with chronic conditions, which is likely to improve QOL [25]. Our findings are consistent with previous reports that showed the success of self-management programs in improving QOL/self-efficacy in Chinese infected with chronic hepatitis B [26] or C [13, 14] and in other hepatitis studies [27, 28].

Regarding the behavioral changes, alcohol avoidance, and stress management also improved in the intervention group. However, unlike the previous reports [11, 29], there were no significant improvements in healthy behaviors such as quitting smoking in the two groups. One possible explanation is that during hospitalization, the healthcare workers might have advised the participants to change their lifestyle, something that might have had such an impact. This explanation stems from the observation that at baseline, participants had high scores regarding their perceived cognition of the disease and there was likely to be less room for them to improve their healthy behaviors during our study period. Moreover, in regard to the lack of improvement in some healthy behaviors, our study population differed from most studies included in the systematic review by Shah and colleagues [11]. We studied usual patients, whereas the review by Shah and colleagues included studies with high-risk behavior patients for whom behavioral changes are more critical and more easily observed. It is also important to note that the UC group significantly decreased their healthy behaviors, suggesting that the educational program was most likely to prevent risk taking behavior in the intervention group.

We used self-management education adjusted to evaluate items used in the questionnaire. As a result, these strategies improved CLDQ scores, confirming that developing an action plan and using problem-solving discussion are essential for self-management education. The effects of the educational program were more significant at 6-month assessment, indicating that the benefits are likely to last longer when the program is conducted for a more extended period of time.

In this study, about 35% of the participants were considered as having self-report symptoms of depression, and this might be an overestimation because reports show that its occurrence among the general Chinese population is about 1.6% [30]. However, the 35% figure might be due to antiviral therapy, whose side effects include depression [7]. Even though the number of participants with depression in the intervention group decreased from 13 to 5, we did not find any significant differences between the intervention and the UC groups probably due to the small sample size. Previous studies found that depression was remarkably reduced after self-management patient’s education [16, 31]. Research has demonstrated that patients with depression are likely to complain about stigma [9] and fatigue that might affect their QOL [32]. Therefore, lack of societal protection from stigmatization has the potential to make depression worse. In our study, we took sufficient time and focused on coping with stress strategies not only during hospitalization but also after discharge. Continuous support from the nurse leads to a feeling of reassurance among the patients, which reduces anxiety and depression [33].

Even though participants were randomly assigned, more females were allocated to the intervention group. There were differences in adherence to medical treatment according to gender; females adhere less as they are influenced by social factors [34]. In this study, including gender as a covariate in ANCOVA analysis had a significant effect on the results, suggesting that the program itself contributed to improved outcomes in this study.

Consequently, more attention needs to be focused on female patients, and an early psychotherapy intervention for patients treated with interferon is justified.

There were different completion rates of behavior recording between the two groups. The intervention group completed the task better. During the phone-call follow-up, nurses applied principles of the self-efficacy theory [35] and enhanced self-confidence to carry out the action plan. For example, the nurse encouraged the patients and congratulated them when they did well. After following the nurse’s advice, the patients’ health-supporting behaviors improved, and as they learned more self-management skills, their confidence increased accordingly. Therefore, this was considered to be one of the reasons for the increase in self-efficacy and the change in patient behavior.

Nurses who are trained in liver diseases, such as chronic hepatitis, in primary care, are not present in most primary care systems [36]. China is not an exception. For example, the general population perceives Chinese primary care services as having low-quality equipment resources [37]. In this context, services in place to address viral hepatitis self-management counseling are likely to be absent in China in primary care settings. Therefore, after discharge, patients are more likely to be without any support, and usually, patients might feel anxiety or express concern for life-adjustment and continue treatment. Therefore, it is very important to establish a relationship of trust between the patients, providers and the educational follow-up system from the hospital where patients are being hospitalized. Through developing a sense of trust, self-management education messages can be offered to give patients hope.

### Study limitations

This study was performed in the context of a small-scale program and used a convenience sample among in-patients in a single hospital which could be a limitation for the generalizability of our results to other Chinese settings and countries. However, our outcomes supported the effectiveness of previous self-management education even in different healthcare systems.

### Implications for practice

This program was found to be highly effective. Therefore, it serves as a reminder for local nurses who care for patients with chronic viral hepatitis to acquire this skill to address the self-management needs of their patients. It also acts as a reminder to the community of nurses that this type of program is feasible and can be implemented for hepatitis patients not only in hospital but also can be continuously embedded in community health. We should consider providing continuous support to patients living with chronic viral hepatitis and regular activities related to health promotion to ensure sustainability beyond this type of intervention.

## Conclusion

This program helped patients with chronic viral hepatitis to acquire self-management skills to cope with their daily illness needs, significantly improved their QOL, and helped the patients to adopt health behaviors such as alcohol avoidance, and stress management. Our findings show that it is feasible to provide a self-management program at both the hospital and community level. Therefore, this type of program has the potential to improve awareness to prevent an acute stage of chronic hepatitis, avoid disease transmission, further deterioration and prevention from hospital readmission. By doing this, we may reduce the economic burden on the patients and the health system. Even though this program was conducted only in one hospital, the findings of this study may serve to inform future studies in other settings in China.

## Additional file

Additional file 1: Is the English language versions of the questionnaires developed and used in this study. (DOCX 38 kb)

## Data Availability

The data supporting the results of this study are not publicly available as protection of personal information is required in this research as stated when participants gave their informed consent. However, the corresponding author is available to discuss any issue related to data requests.

## Abbreviations

BL: Base line
CES-D: The Center for Epidemiologic Depression scale
CI: Confidence interval
CLDQ: Chronic Liver Disease questionnaires
GSES: General Self Efficacy Scale
HBV: Hepatitis B virus
HCV: Hepatitis C virus
I – J: Difference in average value
IV: Intervention group
M: Month
SD: Standard Deviation
UC: Usual care group
