# Supplementary material for: Efficacy of a self-management program in patients with chronic viral hepatitis in China
Source: BMC Nurs. 2019 Sep 18;18:44. doi: 10.1186/s12912-019-0366-7 (PMC6749624; doi:10.1186/s12912-019-0366-7)
Supplement: Supplementary file 1 — Is the English language versions of the questionnaires developed and used in this study. (DOCX 38 kb) [file 12912_2019_366_MOESM1_ESM.docx]

**The cognition to illness and cognition to behavioral change**

**Thank you for agreeing to participate and cooperating in the research. Please read the following contents carefully and draw a ‛○’ on the best item according to your actual situation.**

**1.** **Do you think if you continue this situation, there will be a high probability of becoming severe, such as cirrhosis or hepatocellular carcinoma?**

Disagree completely Disagree Neither Agree Agree completely

|  |  |  |  |
| --- | --- | --- | --- |

1 2 3 4 5

**2．Do you think it is very serious if the disease develops to a severe degree, such as cirrhosis or liver cancer？**

Don’t feel serious Don’t feel serious Neither Serious Very serious

| at all |  |  |  |
| --- | --- | --- | --- |

1 2 3 4 5

**3.** **Do you think it is meaningful to implement self-management behavior to prevent your disease from becoming serious？**

No meaning completely No meaning Neither Meaningful Extremely meaningful

|  |  |  |  |
| --- | --- | --- | --- |

1 2 3 4 5

**4．Do you think it's very difficult for you to implement self-management behavior?**

Very difficult Difficult Neither Not difficult Not difficult at all

|  |  |  |  |
| --- | --- | --- | --- |

1 2 3 4 5

**Health behavior change**

**1．Taking medicine**

| not carried out | carried out sometimes | carried out once a week | carried out 2 to 3 days a week | carried out 4 to 5 days a week | carried out every day |
| --- | --- | --- | --- | --- | --- |
|  |  |  |  |  |  |
| 1 | 2 | 3 | 4 | 5 | 6 |

**2．Dietary habit**

| not carried out | carried out sometimes | carried out once a week | carried out 2 to 3 days a week | carried out 4 to 5 days a week | carried out every day |
| --- | --- | --- | --- | --- | --- |
|  |  |  |  |  |  |
| 1 | 2 | 3 | 4 | 5 | 6 |

**3．Prevention of fatigue**

| not carried out | carried out sometimes | carried out once a week | carried out 2 to 3 days a week | carried out 4 to 5 days a week | carried out every day |
| --- | --- | --- | --- | --- | --- |
|  |  |  |  |  |  |
| 1 | 2 | 3 | 4 | 5 | 6 |

**4．Quitting smoking**

| not carried out | carried out sometimes | carried out once a week | carried out 2 to 3 days a week | carried out 4 to 5 days a week | carried out every day |
| --- | --- | --- | --- | --- | --- |
|  |  |  |  |  |  |
| 1 | 2 | 3 | 4 | 5 | 6 |

**5．Alcohol avoidance**

| not carried out | carried out sometimes | carried out once a week | carried out 2 to 3 days a week | carried out 4 to 5 days a week | carried out every day |
| --- | --- | --- | --- | --- | --- |
|  |  |  |  |  |  |
| 1 | 2 | 3 | 4 | 5 | 6 |

**6．Stress management**

| not carried out | carried out sometimes | carried out once a week | carried out 2 to 3 days a week | carried out 4 to 5 days a week | carried out every day |
| --- | --- | --- | --- | --- | --- |
|  |  |  |  |  |  |
| 1 | 2 | 3 | 4 | 5 | 6 |

**Qualitative evaluation of the program**

Thank you for being participated in this study and for your cooperation. To make this educational program more effective, we will appreciate if you evaluate this program based on your impression that you experienced in this study. Please put “○” on the box/items most suitable for you.

- Overall impression of being participated in this program

Very good Good Neither Not very good Not good

|  |  |  |  |
| --- | --- | --- | --- |

- Program period set in this study（in hospital＋6 months after discharge）

Too long 　　　A little long 　　　 Neither　　　 A little short Too short

|  |  |  |  |
| --- | --- | --- | --- |

- Your impression on nurses’ communication skills on the face-to-face guidance

Very good Good Neither Not very good Not good

|  |  |  |  |
| --- | --- | --- | --- |

- Your impression on nurses’ communication skills on the telephone-based guidance

Very good Good Neither Not very good Not good

|  |  |  |  |
| --- | --- | --- | --- |

- Your evaluation of the contents what you have learned

Very appropriate Appropriate Neither Not very appropriate Not appropriate

|  |  |  |  |
| --- | --- | --- | --- |

- Necessity of this program

Very necessary Necessary Neither Not very necessary Not necessary

|  |  |  |  |
| --- | --- | --- | --- |

**〔The Self-management notebook〕**

- Did you use the notebook?

Frequently used Used Neither Sometimes used Never used

|  |  |  |  |
| --- | --- | --- | --- |

- Did you come to carry and use the notebook?

Daily carried Often carried Sometimes carried Rarely carried Never carried

|  |  |  |  |
| --- | --- | --- | --- |

- Convenience of using the notebook

Very convenient Convenient Neither Not very convenient Not convenient

|  |  |  |  |
| --- | --- | --- | --- |

- Impression of the contents of the notebook

Very useful Useful Neither Not much useful Useless

|  |  |  |  |
| --- | --- | --- | --- |

- Please mark “○” that you think that the contents are good.

　　The way of posting photos　　The contact information section

　　Relevant contents of disease cautions

　　The way of setting behavioral goals 　　Re-examine the daily habits

　　A table that could record the results of the laboratory data

　Other（　　　　　　　　　　　　　　　　）

**〔The Self-management textbook〕**

- Have you read the contents of the textbook?

Read Read Sometimes Not always Never

carefully　 through read read　　 read

|  |  |  |  |
| --- | --- | --- | --- |

- Did you understand the contents of the textbook?

Well understood Understood Neither Not understood Not understood

　 　　 much at all

|  |  |  |  |
| --- | --- | --- | --- |

- Did you think this textbook was valuable to you?

Very valuable Valuable Neither Not very valuable Not valuable at all

|  |  |  |  |
| --- | --- | --- | --- |

- Your impression on the adequacy of the contents of the textbook

Very adequate Adequate Neither Not very adequate Not adequate at all

|  |  |  |  |
| --- | --- | --- | --- |

- Please comments on the self-management textbook and notebook, and nurses' guidance.

Thank you very much for your cooperation.
